# Three-dimensional visualisation and articulating instrumentation: Impact on simulated laparoscopic tasks

**DOI:** 10.4103/0972-9941.41938

**Published:** 2008

**Authors:** James G Bittner, Christopher A Hathaway, James A Brown

**Affiliations:** Virtual Education and Surgical Simulation Laboratory (VESSL), Medical College of Georgia School of Medicine, Augusta, Georgia, USA; 1 Section of Urology, Department of Surgery, Medical College of Georgia School of Medicine, Augusta, Georgia, USA

**Keywords:** 3D visualisation, laparoscopic training, simulation

## Abstract

Laparoscopy requires the development of technical skills distinct from those used in open procedures. Several factors extending the learning curve of laparoscopy include ergonomic and technical difficulties, such as the fulcrum effect and limited degrees of freedom. This study aimed to establish the impact of four variables on performance of two simulated laparoscopic tasks.

**Methods:**

Six subjects including novice (n=2), intermediate (n=2) and expert surgeons completed two tasks: 1) four running sutures, 2) simple suture followed by surgeon's knot plus four square knots. Task variables were suturing angle (left/right), needle holder type (standard/articulating) and visualisation (2D/3D). Each task with a given set of variables was completed twice in random order. The endpoints included suturing task completion time, average and maximum distance from marks and knot tying task completion time.

**Results:**

Suturing task completion time was prolonged by 45-degree right angle suturing, articulating needle holder use and lower skill levels (all *P* < 0.0001). Accuracy also decreased with articulating needle holder use (both *P* < 0.0001). 3D vision affected only maximum distance (*P*=0.0108). For the knot tying task, completion time was greater with 45-degree right angle suturing (*P*=0.0015), articulating needle holder use (*P* < 0.0001), 3D vision (*P*=0.0014) and novice skill level (*P*=0.0003). Participants felt that 3D visualisation offered subjective advantages during training.

**Conclusions:**

Results suggest construct validity. A 3D personal head display and articulating needle holder do not immediately improve task completion times or accuracy and may increase the training burden of laparoscopic suturing and knot tying.

## INTRODUCTION

Laparoscopy requires learners to develop distinct technical skills from those used in open procedures. The development of this special skill set is responsible for the well-described learning curve for advanced laparoscopy.[1–2] A prolonged learning curve for laparoscopy can be costly in terms of time, resources and patient morbidity.[3] Several factors extending the learning curve include ergonomic and technical difficulties, such as the fulcrum effect and limited degrees of freedom. These factors along with the loss of 3-dimensional (3D) visualisation impose a barrier to expedient technical skills acquisition.

As more complex laparoscopic procedures are performed, the need for instrumentation that improves dexterity (degrees of freedom) in an ergonomic manner becomes important. Robotic surgery offers a technical solution to this problem, although system cost and other requirements limit its widespread use.[4] Alternatively, less expensive hand-held articulating laparoscopic instruments provide seven degrees of freedom and closely mimic human wrist movements, plus offer haptic feedback.

The loss of 3D visualisation and use of fixed monitors requires adaptation to learned cues within a 2-dimensional (2D) environment. Although the loss of depth perception eventually can be overcome with experience, novice and intermediate trainees may note improved laparoscopic task completion time and accuracy using 3D visualization. Ergonomic benefits related to the use of personal head displays (PHD) may lead to reduced operator strain or fatigue and improved overall satisfaction compared to conventional systems.[5–6]

This study aimed to establish the impact of suturing angle (left or right), needle holder type (standard or articulating) and visualisation (2D or 3D) on selected performance measures after two simulated laparoscopic tasks. Additionally, participants completed descriptive evaluations of tested devices at the conclusion of the study to determine subjective effects on task performance.

## MATERIALS AND METHODS

### Subjects

This prospective study was approved by the Institutional Review Board, Medical College of Georgia. All subjects signed informed consent then completed a questionnaire to verify eligibility and prior experience. Demographics and inclusion criteria are listed in Table 1.

**Table 1:** Subject demographics and inclusion criteria

Skill level	Subject description	Laparoscopic dxperience
Novice (n=2)	PGY-2 urology residents	<10 cases
Intermediate (n=2)	PGY-3/4 urology residents	10 - 50 cases
Expert (n=2)	Laparoscopically-trained urology faculty	>200 cases
		
Inclusion criteria		
Right-hand dominant		
Normal or corrected-to-normal visual acuity in both eyes		
No know color blindness		
No know depth perception disorder		
No know neurologic or motor dysfunction disorder		
No prior training with or use of 3D visualization during laparoscopic surgery		
No prior training with or use of hand-held laparoscopic articulating needle drivers		

### Materials

The Autonomy Laparo-Angle Needle Holder (Cambridge Endoscopic Devices Inc, Framingham, MA) is a hand-held laparoscopic instrument designed to facilitate suture passage and knot tying. This disposable device is composed of five functional parts, including the needle holder jaws on the end of a 5-mm shaft, proximal and distal bending sections, locking mechanism, axial rotation knob and jaw actuation lever. The device exploits the fulcrum effect intrinsic to laparoscopy in order to transfer the hand movements of the operator, through the proximal and distal bending sections, to the needle holder tip. To facilitate needle grasping, the distal section bends approximately 90 degrees with seven degrees of freedom for tip positioning. The needle holder tip also rotates 360 degrees around the instrument axis regardless of the bending angle using the axial rotation knob.

The EndoSite 3Di Digital Vision System (Viking Systems Inc, La Jolla, CA) is an FDA-approved comprehensive 3D visualisation system designed to provide minimally invasive surgeons an immersive operative experience. The advanced system used in this study included a 10 mm, 0-degree high-definition (HD) stereoscope connected to a 300-watt xenon light source. The stereoscopic images are transmitted through dual video output channels directly to a data processing unit (ViPort). The ViPort converts both video signals to HD-SDI and delivers real-time clinical images to the surgical team via the PHD. In turn, the PHD provides 3D-HD immersive vision by way of liquid crystal display panels for each eye. The PHD is lightweight and fully adjustable to ensure optimal comfort and viewing angle for all users. The EndoSite 3Di system also provides voice-activated, picture-in-picture technology for instant access to existing diagnostic imaging studies or live secondary video.

Materials used for all simulated laparoscopic tasks included 3-0 Vicryl on SH needle (Ethicon Endo-Surgery, Cincinnati, OH) cut to 12-cm for suturing and knot tying and 9-cm long penrose drains (Taut Inc, Geneva, IL). Along the long axis of the penrose drains a total of four dot pairs were created in a standardized fashion 2-cm apart and separated by 1.5-cm in the longitudinal plane. Next a 6-cm vertical slit was created in the drains directly between horizontal dot pairs. Subjects used a new penrose drain for each exercise to ensure the same haptic feedback with regard to needle puncture and suture passage. The penrose drains were grasped on either end by an alligator clip attached to opposite corners of a square board (SimuLab Corporation, Seattle, WA). To avoid axis bias and mimic urethrovesical anastomosis, the angle of the sutured object (penrose drain) was altered between left and right 45-degree angles along the coronal plane.[7–8]

To facilitate correct ergonomics, several aspects of the setup were controlled. The position of the square boards inside the height-adjustable laparoscopic box trainer remained constant. Participants held the instrumentation with the shoulder adducted, ensuring the elbow formed a right angle. Instruments were introduced through the same port sites for all tasks. Ergonomic HiQ+ laparoscopic needle holders (Olympus Surgical America orangeburg, NY) were used in the non-dominant hand for all tasks. In addition, the stereoscope was secured to the box trainer to ensure standardised view orientation and magnification. For tasks completed without the PHD, the 3D-HD camera was switched to 2D-HD mode with images projected on an HD video monitor (Olympus Surgical America orangeburg, NY). The monitor was positioned at 10-degrees upward and 30-degrees leftward gaze of the box trainer, mimicking a traditional operating room setup. Gaze for the PHD was approximately frontal and downward in the direction of the operator's hands.

### Study Protocol

Device orientation, skills practice and simulated laparoscopic task performance occurred during a single day in the Virtual Education and Surgical Simulation Laboratory (VESSL). Prior to actual task performance, all participants completed 30 minutes of monitored suturing and knot tying practice with the articulating needle driver in an open laparoscopic trainer. Experience with the PHD was not permitted.

Subjects completed two distinct laparoscopic tasks: 1) running sutures, 2) simple suture followed by surgeon's knot plus four square knots. These tasks require suture handling, dexterity, depth perception and proper pronation/supination to complete the objective. Task variables included the suturing angle (left or right), needle holder type (standard or articulating) and visualisation (2D or 3D). Each task with a given set of variables was completed twice in random order. A single individual recorded all task completion times, distance measurements and knot security. Observation of subject performance in real-time from the operator's point-of-view was facilitated by the 2D-HD video monitor connected to the laparoscopic system. At the conclusion of the study, each participant completed an independent evaluation of the Autonomy Laparo-Angle Needle Holder and EndoSite 3Di Digital Vision System.

### Suturing Task

Subjects grasped and oriented the needle intracorporeally then completed four running sutures in a penrose drain. The direction of task execution (suturing toward the operator and right to left) remained constant and knot tying was not permitted during this exercise. Participants were evaluated for accuracy based on the maximum distance the suture passed from any one black dot and the average distance across all dots. Each event was timed from first needle touch to suture visualisation following the last stitch.

### Knot Tying Task

Participants grasped and oriented the needle intracorporeally, placed a single stitch through dots on a penrose drain, then performed an intracorporeal surgeon's knot followed by four square knots. Objective evaluation included time to complete the task, accuracy based on maximum and average distance from the dots and knot security. Each exercise was timed from first needle touch to cutting of the suture following the last knot in the same method as previously described. Sutures were cut by participants using HiQ+ laparoscopic Metzenbaum scissors (Olympus Surgical America orangeburg, NY).

### Statistical Analysis

The endpoints of interest included suturing and knot tying task completion times, plus average and maximum distance from specified marks. The predictor variables were suturing angle (left or right), needle holder type (standard or articulating), visualization (2D or 3D) and skill level of participants (novice, intermediate, expert). Repeated measures two-way analysis of variance (ANOVA) using mixed models was employed to examine recorded endpoints. A Bonferroni adjustment was used prior to pair-wise post hoc analyses. Data are presented as mean and standard deviation. All statistical significance was assessed at alpha level 0.05 using SAS 9.13 (SAS Institutes Inc, Cary, NC).

## RESULTS

### Suturing Task Completion Time

This study demonstrates relationships within three of four variables on suturing task completion times including suturing angle, needle holder type and skill level of participants. Right 45-degree angle suturing, articulating needle holder use and lower skill levels all prolonged suturing task completion times (*P* < 0.0001; [Table 2]). Left 45-degree suturing decreased task time by 18% across all groups (254 ± 111 sec *vs.* 209 ± 102 sec). No differences were noted between 2D and 3D visualization.

**Table 2:** Impact on suturing task completion time and accuracy

Effect*	Mean time (sec)	F value	Significance†
Suturing angle	Left = 209		
	Right = 254	18.46	<0.0001
Needle holder	Std = 156		
	Artic = 300	173.97	<0.0001
Visualization	2D = 225		
	3D = 238	1.61	0.2083
skill level	Novice = 265	17.7	<0.0001
	Intermed = 240		
	Expert = 189		
**Effect**	**Average distance (mm)**	**F value**	**Significance**
Suturing angle	Left = 0.7		
	Right = 0.8	2.27	0.1357
Needle holder	Std = 0.5		
	Artic = 1	66.2	<0.0001
Visualization	2D = 0.7		
	3D = 1	2.75	0.1012
Skill level	Novice = 1		
	Intermed = 0.7		
	Expert = 0.6	6.63	0.0021
**Effect**	**Maximum distance (mm)**	**F value**	**Significance**
Suturing angle	Left = 1.7		
	Right = 1.9	2.15	0.1467
Needle holder	Std = 1.1		
	Artic = 2.3	64.61	<0.0001
Needle holder	Std = 1.1		
	Artic = 2.3	64.61	<0.0001
Skill level	Novice = 2		
	Intermed = 1.5		
	Expert = 1.8	3.84	0.00254

* Suturing angle (left / right), needle holder (standard / articulating), visualization (2D / 3D), skill level (novice / intermediate / expert)

† P value from repeated measures analysis of variance with mixed effects (alpha=0.05)

Within the novice level, participants took longer to complete the task when using the articulating needle holder compared to a standard instrument regardless of suturing angle (344 ± 88 sec *vs.* 165 ± 42 sec for right and left, respectively) or visualisation type (366 ± 72 sec *vs.* 184 ± 35 sec for 2D and 3D, respectively). Suturing task time did not change significantly when switching from 2D to 3D visualisation.

Within the intermediate group, trainees needed more time when left-angle suturing with the articulating needle holder in 2D compared to the same task using a standard needle holder (307 ± 170 sec *vs.* 157 ± 76 sec; *P* < 0.0001). Right-angle suturing combined with articulating needle holder and PHD appeared to be most difficult for intermediate trainees. Under these conditions, they performed right-angle suturing significantly slower than left-angle suturing (389 ± 152 sec *vs.* 219 ± 19 sec; *P* < 0.0001).

Overall experts completed the suturing task in the least amount of time. Within this group, subjects required longer to complete the task when using the articulating compared to standard needle holder (258 ± 68 sec *vs.* 122 ± 12 sec; *P*=0.0003). Experts used the articulating needle holder more quickly to complete the task compared to novices despite suturing angle and visualisation (258 ± 68 sec *vs.* 340 ± 70 sec; *P* < 0.0001). When right-angle suturing with the articulating needle holder in 2D, experts used significantly less time than intermediate trainees (264 ± 89 sec *vs* 241 ± 60 sec; *P*=0.0001).

### Suturing Task Accuracy

Both needle holder type and lower skill level individually increased the average distance from specified dots on the penrose drains [Table 2]. Across suturing angle, visualisation and skill level, articulating compared to standard needle driver use resulted in greater average distance from the dots (*P* < 0.0001). Novice participants were the least accurate overall and in this group the articulating needle holder further decreased accuracy by 100% and 130% with 2D and 3D vision, respectively. The intermediate and expert participants also exhibited greater average distances from dots on the penrose drains in 3D mode. Accuracy measured by average distance from dots, when all groups were pooled, was not affected by either suture angle or visualisation type.

Maximum distance from specified dots was affected by needle holder type, visualisation and skill level [Table 2]. Post hoc evaluations demonstrate several specific differences in performance. In 2D the articulating needle holder resulted in 34% longer maximum distance from specified dots compared to standard instrumentation (1.9 ± 0.2 mm *vs.* 1.3 ± 0.2 mm; *P*=0.0028). 3D visualization exacerbated the differences between use of standard and articulating needle driver use (*P* < 0.0001). When experts used the articulating needle driver, maximum distance increased by 29% in 2D and 218% in 3D. With respect to maximum distance, all subjects demonstrated the most difficulty with right-angle suturing in 3D (*P*=0.0017). There was no significant effect of suture angle on accuracy when all groups were pooled. All variables combined, 3D compared to 2D visualisation decreased accuracy by 24% as measured by maximum distance from dots.

### Knot Tying Task Completion Time

Analysis exposed differences within suture angle, needle holder type, visualisation and skill level on knot tying task completion time [Table 3]. Right angle suturing significantly increased task time (157 ± 23 sec vs. 148 ± 25 sec, *p*=0.0015) as did the 3D PHD (157 ± 27 sec vs. 148 ± 21 sec, *P*=0.0014). Articulating needle holder use significantly prolonged knot tying time across all skill levels when using 2D visualisation. For example, completion times were increased in the novice (18%), intermediate (24%) and expert (18%) skill levels. In addition, experts performed the task significantly faster than novice trainees (*P*=0.0003). Knots were subjectively more secure when using the 3D PHD visualisation. The average and maximum distances from dots were not significantly different across all variables.

**Table 3:** Impact of variables on knot tying task completion time

Effect*	Mean time (sec)†	F value	Significance†
Suturing angle	Left = 148		
	Right = 157	10.7	0.0015
Needle holder	Std = 136		
	Artic = 143	149.24	<0.0001
Visualization	2D = 148		
	3D = 157	10.9	0.0014
Skill level	Novice= 159		
	Intermed = 154		
	Expert = 145	8.96	0.0003

* Suturing angle (left / right), needle holder (standard / articulating), visualization (2D / 3D), skill level (novice / intermediate / expert)

† *P* value from repeated measures analysis of variance with mixed effects (alpha=0.05)

### Device Evaluations

All participants felt the Autonomy Laparo-Angle Needle Holder required significantly more time to master than a single day experience provided. Five of six subjects (83%) felt the ergonomics of the device were unfamiliar compared to standard laparoscopic instrumentation. Of those, four believed the unusual ergonomics may decrease utility. One expert surgeon and one intermediate trainee (33%) stated that articulating needle holder benefits included multiple degrees of freedom and ease of intracorporeal knot tying.

According to all subjects, the EndoSite 3Di Digital Vision System provides excellent high-definition 3D graphics by way of an easy-to-use, lightweight PHD. All participants save one expert surgeon (83%) felt that 3D-HD visualisation may confer some advantage to novice trainees during their introduction to laparoscopic suturing and knot tying tasks. Five of the six (83%) stated they perceived greater depth perception with the PHD. No subject described muscle strain or fatigue, headache or visual disturbances.

## DISCUSSION

Since 1994 when Kavoussi and colleagues first described their clinical experience with robot-assisted laparoscopic cholecystectomy and bladder suspension, multiple reports detailing the use of robots in laparoscopic urologic surgery have been published.[4,9] Interest in this technology stems from the limitations of standard laparoscopic surgery. The ergonomic, psychometric and visual restrictions inherent to laparoscopic surgery include problematic geometry, limited range of motion (four degrees of freedom) and 2D visualisation.[10–12]

When using fixed laparoscopic instruments, oblique directions of suturing are often required. Results of this study suggest a difference exists between suturing angles as related to suturing and knot tying task completion times. Interestingly, accuracy was not affected by suture angle, which suggests that accuracy was maintained at the expense of task completion time. For right-handed surgeons, right-angled suturing resulted in prolonged task completion times compared to left 45-degree suturing. If anything, the increased difficulty of right 45-degree angle suturing should have favoured the articulating needle holder, which permits normal 90-degree angulation between needle and needle holder and eliminates the need for wrist rotation during suture passage.

The limited range of motion with standard laparoscopic instruments may also influence performance by decreasing surgeon dexterity.[13] The device used in this study provided seven degrees of freedom, but notably prolonged suturing and knot tying task completion times across all skill levels. Studies comparing standard laparoscopic instruments to robotic devices showed task completion times were significantly shorter with standard laparoscopic instrumentation. Alternatively, the robotic system seemed to yield more precise performance. Prolonged times with robotic systems were attributed to the learning curve required for articulating instrumentation and 3D visualisation.[13–14] 

One reason for the difficulty experienced by subjects in the current study may be the steep learning curve associated with hand-held articulating instrumentation. This is supported subjectively by all participants who felt that 30 min of mentored practice and 35 to 45 min of task performance time were insufficient to overcome the learning curve of the articulating needle holder. Five of the six subjects (83%) felt the ergonomics of the device were unfamiliar compared to standard laparoscopic instrumentation. If participants were allowed sufficient time to surmount the learning curve prior to skills performance, task completion times using the articulating device may be more comparable to standard laparoscopic equipment.

Another issue crucial to minimally invasive surgery is limited stereoscopic visualisation. Laparoscopic experts rely on 2D visual cues including shading, texture gradient, familiar anatomy, movement of the laparoscope and structure size to assure proficient and precise movements.[12] The 3Di system has been described as comfortable due to improved ergonomics and useful in transanal endoscopic microsurgery and multiple neurosurgical procedures.[15–18] Not surprisingly, the present investigation showed that experts completed the simulated advanced laparoscopic suturing and knot tying tasks in less time and did so more accurately than intermediate or novice subjects. Since novice trainees do not posses the experience to depend on 2D cues, which may result in depth perception errors and iatrogenic injury, it was anticipated that this group might benefit from 3D visualisation.[19] Visualisation impacted suturing and knot tying task completion times and suturing task maximum distance, although it did not affect average distances for either simulated task. Further analysis revealed the PHD slightly delayed knot tying completion time compared to 2D, but the resultant knots were subjectively more secure. All novice trainees felt that the 3D vision conferred some advantage during their introduction to laparoscopic suturing and knot tying and provided greater depth perception than 2D-HD monitor display.

Jones and colleagues studied the impact of 3D visualization on novice, intermediate and expert surgeon performance of five simulated laparoscopic tasks, including suturing and intracorporeal knot tying. In their study, experts performed significantly faster than novices on all tasks regardless of visualisation. Novices benefited slightly more than intermediate trainees while experts showed no benefit with 3D. Subjectively, 30 participants (60%) perceived more instrument control and performance accuracy in 3D.[20] With regard to intracorporeal suturing, other authors claim there is no significant influence of 3D vision on expert task completion time or suture placement.[21] We found slight impairment of training task parameters (maximum distance and time) when 3D vision and the articulating needle driver were employed.

Using similar inclusion criteria to our own, one study required novice trainees to complete three basic simulated laparoscopic tasks comparing 2D to 3D visualisation. There was no difference in completion times; yet, 68% of users felt the 3D PHD provided better overall view than the 2D systems.[22] Other investigators have compared a 2D video monitor system to different 3D systems, including the precursor to the PHD used in the current study. Overall user performance was 12% slower in the 3D group, but the PHD increased task accuracy in both groups.[23]

Bhayani and Andriole determined that minimally invasive surgeons should consider 3D visualisation for learning advanced laparoscopic procedures. In their investigation, novices completed a basic laparoscopic task using either a 2D monitor or 3D PHD. Data confirmed mean performance time was significantly faster using 3D visualisation and 58% of users preferred the 3D system. Investigators surmised that novices might progress to experts more rapidly if 3D PHD was used during initial training.[5] Patel conducted a study utilising novice and expert subjects who completed five simulated complex laparoscopic tasks once each using a 2D monitor then the PHD. The authors suggest that 3D PHD offers some acceleration of the learning curve for novices with regard to precise and timely achievement of complex laparoscopic tasks [Table 4].[24]

**Table 4:** Recent studies of three-dimensional (3D) compared to two-dimensional (2D) visualization

Study (reference)	3D device	Laparoscopic tasks	Validity type	Outcomes
Jones, 1996[15]	3D monitor	Simple suture and instrument tie	Construct	12% faster performance in 3D; 60% perceived more instrument control and accuracy in 3D
Herron, 1999[17]	3D monitor; 3D PHD*	Rope pass, cup drop, triangle transfer	Construct	No difference in task performance time using 2D or 3D; 68% felt 3D PHD provided better overall field of view
Hanna, 2000[16]	3D monitor	Running suture closure of enterostomy	None	No difference in time, closure integrity or suture accuracy between 2D and 3D; 0% expressed difference in depth perception
Cheah, 2001[24]	3D PHD	Instrument tie	None	10% slower performance in 3D
Thomsen, 2004[18]	3D PHD	Stereoacuity test	None	12% slower performance in 3D
Bhayani, 2005[5]	3D PHD	Bead transfer	None	18% faster performance in 3D; 58% preferred the 3D PHD
Maithel, 2005[6]	3D PHD	Triangle transfer	Construct	8% better motion smoothness with 3D PHD; 66% junior residents preferred the 3D PHD
Lin, 2006[23]	3D PHD	Virtual diathermy	None	No difference in task performance, gaze angle or fatigue
Patel, 2007[19]	3D PHD	Cutting, suturing, instrument tie	Construct	Improved accuracy and decreased error rates in 3D; 100% described better depth perception and structure definition
Byrn, 2007[10]	Robot	Bead transfer, threading, cap, instrument tie	None	Faster task performance and decreased error rates in 3D independent of articulating instrumentation

* PHD - Personal Head Display

The limitations of the current study include the small number of participants and the relatively short training time permitted with each tested device. Training with the articulating needle holder to a level of competence equivalent to standard instrumentation before task performance might have impacted results. It is conceivable that extending the mentored training period with each device prior to skills assessment may yield shorter task completion times and improved accuracy. Statistical impact on task performance was noted with each of the two devices in a laboratory setting but the study was not designed to suggest these differences are reproducible under clinical conditions. Larger studies designed with these limitations in mind are needed to determine the impact of new laparoscopic tools on training time and ultimately clinical outcomes.

## CONCLUSIONS

The simulated complex laparoscopic tasks performed in this study suggest construct validity. The Autonomy Laparo-Angle Needle Holder prolonged simulated laparoscopic suturing and knot tying task completion times and decreased accuracy across all variables. In addition, right 45-degree angle suturing increased laparoscopic task completion times. Only in combination with the articulating needle holder was there prolonged knot tying task completion time and increased maximum distance using the EndoSite 3Di Digital Vision System. Although the majority of subjects perceived greater depth perception with the 3D PHD compared to 2D visualisation and felt the technology conveyed an advantage for novice trainees, the 3Di system did not improve overall task performance.
